# Supplementary material for: Are carbon emissions trading and green financial instruments synergistic? -Comprehensive quantitative research based on content analysis
Source: PLoS One. 2024 Mar 7;19(3):e0298601. doi: 10.1371/journal.pone.0298601 (PMC10919601; doi:10.1371/journal.pone.0298601)
Supplement: S1 Data — (ZIP) [file pone.0298601.s001.zip › Data/地级市Z_F工作报告.zip!/╣π╢1⁄2/╤⌠╜¡╩╨/└┤╫╘╣1⁄2╓┌║┼í╛┬φ┐╦╩2╛▌═°í┐_2009.docx]

各位代表：

　　我代表市人民政府向大会作政府工作报告，请予审议，并请政协各位委员和其他列席人员提出意见。

2008年工作回顾

　　2008年是极不平凡的一年，是我市经受住冰冻寒冷、“黑格比”强台风等自然灾害和国际金融风暴的严峻考验，科学发展取得重要成绩的一年。一年来，在省委、省政府和市委的正确领导下，在市人大及其常委会和市政协的监督支持下，市政府深入贯彻落实科学发展观，继续解放思想，坚持改革开放，以工业为主攻方向，以招商引资为突破口，以科技创新为动力，壮大经济综合实力，提升民生质量，推进文化创新，着力打造电力能源基地、“双转移”示范地、旅游休闲度假胜地、宜居创业滨海新城，经济保持平稳较快发展，社会各项事业全面进步，较好地完成了市五届人大三次会议确定的目标任务。预计2008年全市实现生产总值498.0亿元，增长12.5%；人均生产总值20479元，增长10.7%；地方财政一般预算收入16.36亿元，增长26.1%；全社会固定资产投资170.8亿元，增长26.8%。全市金融机构人民币存款余额391.9亿元、贷款余额158.4亿元，分别比年初增长24.2％和16.8%。单位生产总值和单位工业增加值能耗分别下降3.5%和12.6%。节约用地取得新成效。

　　——工业发展步伐加快。出台实施了一系列保持经济增长的政策措施，深入开展“工业企业集中服务月”活动，工业保持较快增长。全市规模以上工业总产值445.9亿元，增加值105.9亿元，分别增长20.4%和19.6%。阳江核电站正式动工，广东华厦阳西电厂建设加快，500千伏蝶岭输变电工程投入运行。产业转移工业园建设取得新突破，广州（阳江）产业转移工业园竞争成为省示范性产业转移园。华讯电子、和兴金属制品等一批企业建成投产。五金刀剪、食品饮料和机械电器等重点行业呈现平稳增长态势。

　　——农业经济稳步增长。全面落实各项惠农政策，农业综合生产能力进一步提高。全市农业总产值190.5亿元，增长3.0%。农业结构调整持续优化，特色经济作物种植规模进一步扩大。渔业在遭受强台风“黑格比”重创的情况下仍实现平稳增长，渔业经济总产值95.0亿元，增长9.5%，水产品出口总值和增速均居全省第二。东平渔港被认定为国家级中心渔港。市谊林海达速冻水产有限公司被评为国家重点农业龙头企业，实现我市国家级农业龙头企业零的突破。畜牧业稳步发展。全市畜牧业总产值40.7亿元，增长4.7%。林业取得新成绩，全市完成造林面积11.1万亩。农田基本建设加快，整治农田面积5.9万亩。水利防灾减灾工程、病险水库除险加固工程、农村饮水安全工程建设有新进展，水毁水利工程抢险修复进展顺利。

　　——现代服务业蓬勃发展。出台鼓励消费的政策措施，扶持发展现代服务业。第三产业增加值172.9亿元，增长12.9%。社会消费品零售总额265.6亿元，增长19.6%。旅游业发展水平不断提升，荣获“中国最佳生态旅游城市”、“中国温泉之乡”、“最佳休闲城市”等称号。全市接待游客562.7万人次，旅游总收入30.0亿元，分别增长5.1%和7.1%。房地产业健康发展，实施了一系列促进房地产业稳定发展的政策措施，全市房地产开发投资21.2亿元，增长23.3%。批发零售、住宿餐饮、交通运输、邮电通信、商贸物流、商务会展等快速增长。江城区城北街道南湾社区成为全市首个“全国商业示范社区”。

　　——城乡面貌变化喜人。城乡规划管理有效加强，完成了广州（阳江）产业转移工业园、市政公共配套设施、近百村庄和一批重要区域的规划编制。城市建设强力推进。高标准改造了东风一、二路和北环路，城南新区江朗大道建成通车。市第一净水厂扩建工程顺利完成，日处理能力从2万吨提高到5万吨；阳东县、阳西县污水处理厂投入试运行。奕垌垃圾场二期扩建工程建设基本完成。城乡基础设施建设全面加快。云阳高速公路阳阳段、开阳高速公路那龙出口至阳江核电站公路一期工程动工建设，阳茂高速公路织立交至阳西县城连接线一级公路和S113线南段二期工程、阳江港疏港大道等一批公路建成通车，镇通村公路硬底化建设完成302公里。无电村通电工程按期完成。城市管理水平明显提高。市区市容市貌整治行动和市区道路交通安全综合治理全面开展，城市环境有了很大的改观，顺利通过广东省卫生城市复检。国土资源管理进一步加强，连续十年实现耕地占补平衡。建立健全了土地储备制度，政府调控土地一级市场力度明显加大。积极盘活存量土地，依法清理处置闲置土地，全市累计处置闲置土地182宗313.21公顷。环境保护成效显著。环境保护责任制进一步落实，环境综合整治有效加强，节能减排工作取得新进展，在全省环境保护责任考核中连续五年获得优秀等次。

　　——开放型经济有效推进。完善了《阳江市招商引资奖励办法》，全市各级招商引资积极性进一步提高。选派8个小分队到珠三角地区全方位开展驻点招商，主动承接产业转移。成功举办第七届中国（阳江）国际五金刀剪博览会暨第八届阳江市旅游文化节，积极组团参加国内外大型经贸活动。全年实际吸收外商直接投资145亿美元，增长10.8%。外贸进出口总额12.5亿美元，增长7.5%，其中外贸出口总额11.3亿美元，增长6.2%，利用外资和外贸出口工作均获得省的表彰。

　　——各项改革继续深化。行政体制改革稳步推进。按照统一、精简、高效的原则，调整理顺阳江高新技术产业开发区管理体制，实行区镇港合一的管理模式，为高新区加快发展奠定了良好基础。制订了岗美华侨农场改革与发展方案，积极推进场办社会分离工作。推动综合行政执法体制改革，调整理顺市直交通、城市管理综合执法机构设置，执法行为进一步规范。加快事业单位改革，规范行政事业单位人员录用制度。投融资体制和国有企业改革深入推进。推进了阳春轴承公司产权转让和市国营电瓷厂、华月陶瓷公司破产以及部分国有企业改制工作。农村信用社改革试点工作顺利完成。华龙集团破产重组后恢复上市交易。其他各项改革稳步推进。

　　——社会事业全面进步。自主创新能力不断增强。组建国家刀剪及日用金属工具质量监督检验中心和五金刀剪省部产学研创新联盟，成功创建“国家火炬计划阳江新型功能刀剪材料设计与先进制造产业基地”，全年新增中国驰名商标3件、省著名商标19件，中国出口免验企业1家，省名牌产品11个；创建国家级高新技术企业9家，省级工程技术研发中心1家；专利申请量保持粤西领先地位，获得“全国专利执法先进单位”称号。教育事业加快发展。改扩建中小学校121所，新建学校15所。高考上线人数首次突破万人大关。市技工学校顺利通过国家级重点技工学校评估。阳江职业技术学院办学条件不断改善。出台实施了《阳江市加快引进人才的若干规定》等一系列政策措施，人才工作机制逐步健全。公共卫生三大体系建设进一步加强。市第三人民医院等4所医院改造成为社区卫生服务中心。连续五次荣获“全国无偿献血先进城市”称号。新型农村合作医疗参合率达99.1％，参合人数175.7万人。文体事业稳步推进，广东海上丝绸之路博物馆建设进展顺利。市图书馆、市群众艺术馆完成搬迁并全面对外开放。与河南省开封市缔结为友好城市。市区有线电视数字化整体转换工作基本完成。“全民健身与奥运同行”系列群众性体育活动有序开展，成功举办了市第九届体育节和第三届妇女运动会。人口与计划生育工作取得明显成效。全国婚育新风进万家活动示范市建设稳步推进，全市低生育水平保持稳定。国防动员、兵员征集、民兵预备役和双拥工作进一步加强，再次被评为全国双拥模范城和广东省双拥模范城。民族宗教、妇女儿童、外事侨务、民政、统计、人防、打私、气象、地震、市志、档案、爱卫等各项工作均取得新成绩。

　　——和谐阳江建设成效明显。劳动就业工作进一步加强。全市免费培训劳动力2.4万人次，新增转移农村劳动力6.45万人，城镇新增就业人员4.26万人，帮扶城乡就业困难人员实现就业4046人，零就业家庭保持动态消零，城镇登记失业率控制在2.9％。社会保险覆盖面持续扩大，其中城镇居民医疗保险参保人数39万人，城镇职工基本养老、医疗、失业、工伤、生育保险参保人数达到83.8万人次，增加8.5万人次。城乡最低生活保障制度进一步落实，两次提高了低保对象补助水平，完成9所敬老院改扩建工程。全年改造农村泥砖住房2993户，完成水上渔民安居工程266户，强台风“黑格比”袭击造成的1424户全倒户全部在春节前入住新房。红十字会工作扎实推进，救灾、救助、救护实力增强。支援四川地震灾区救灾重建工作深入开展。城乡居民生活水平稳步提高，城镇居民人均可支配收入11933元，增长7.3％；农民人均纯收入5152元，增长6.5％。居民消费价格总水平累计上涨4％，比全省平均涨幅低1.6个百分点。社会治安综合治理成效显著。110指挥大厅改建和扩容工程投入运行，视频监控系统一期工程建设顺利完成，治安防控联动快速反应机制进一步完善，各类案件持续下降，群众满意度大幅提高。产品质量和食品安全整治取得实效。安全生产目标管理责任制有效落实，安全生产主要控制指标明显下降，安全生产形势保持稳定。人民调解和信访工作不断加强，群体性事件明显减少，人民内部矛盾纠纷得到有效调处和化解，社会保持和谐稳定。

　　——政府自身建设和民主法制建设不断加强。出台了一系列加强政府管理的规章制度，政府依法行政、驾驭市场经济、统筹经济社会发展的能力不断提高。行政集中服务工作有效加强，市直48个单位共262项审批和服务项目在大厅实行集中审批、集中服务，所有审批事项办理时间实现“双减半”，办事效率和服务水平明显提高。坚持向人大及其常委会报告工作、向政协通报情况制度，自觉接受法律监督和民主监督。加强与各民主党派、工商联、无党派人士和工会、共青团、妇联等组织的联系，广泛听取意见。认真做好人大建议和政协提案办理工作，全年办理人大建议99件，政协提案152件、建议案1件。普法宣传教育广泛开展，群众法律意识不断增强。群众性精神文明创建活动广泛开展，阳春市合水镇和广东电信阳江分公司分别被评为全国文明镇和文明单位。村（居）委会换届选举全面完成，村（居）民自治制度不断健全。政务、厂务、村务公开工作取得新成果。廉政建设得到加强，监察审计工作扎实推进，查处违纪违法案件、纠正损害群众利益不正之风工作成效明显。

　　与此同时，我们清醒地看到，我市经济社会发展和政府工作中还存在不少问题，主要表现在：经济总量仍然偏小，税源相对不足，财政收支矛盾突出；产业结构不尽合理，产业集聚度较低；招商引资成效仍不明显，产业园区建设进展未如理想；工业企业规模普遍偏小，自主创新能力较弱，应对风险能力不强；国企改革进展缓慢，历史遗留问题解决压力较大；农业基础薄弱，农业增效和农民增收不明显；各级政府统筹发展能力有待提高，劳动就业、教育卫生、防灾减灾等方面还有不少问题亟待解决；公务员队伍建设仍需加强，部分政府工作人员能力素质、工作作风、精神状态与新形势新任务还不相适应等。我们一定要保持清醒的头脑，增强忧患意识，不断解决发展中突出的矛盾和问题，更加努力做好各项工作，决不辜负人民的信任和期望。

　　各位代表，过去的一年，挑战非同寻常，成绩来之不易。我市发展中取得的每一项进步，都是省委、省政府和市委正确领导的结果；是各级人民代表大会及其常委会和人民政协监督支持的结果；是全市人民克难奋进、开拓进取的结果。在此，我代表市人民政府，向全市人民，向为阳江发展作出贡献的各位人大代表、政协委员、各民主党派、工商联和社会各界人士，中央和省驻阳江的单位，驻地解放军、武警官兵，以及所有关心支持阳江发展的港澳台同胞、海外侨胞和国际友人，表示崇高的敬意和衷心的感谢！

2009年工作安排

　　2009年，是我市进一步贯彻落实科学发展观、推进经济社会平稳较快发展的关键一年。当前，国际金融危机的影响仍然存在，经济运行的困难增加，发达国家和地区对外投资增速不断下滑，产业转移更加慎重，这将对我市经济发展造成一定影响。但我们应该看到，我市经济社会发展具有很多有利条件。国家实施积极的财政政策和适度宽松的货币政策，出台了以扩内需、促发展、保增长为核心的10项提振经济政策；省推出了刺激经济发展的16项措施，大力支持基础设施建设、产业转型升级和民生事业发展，同时实施“双转移”战略。这将有利于推动我市加快发展。我们更要看到，经过20年的建设和发展，我市经济社会发展的基础日渐厚实，自我发展的能力显著增强，资源、区位、环境、劳动力等优势进一步显现，而且阳江核电站、阳阳高速等一批重点项目建设正全面推进，必将强力拉动我市经济社会发展。因此，我们要认真研究分析国内外经济形势对我市的影响，用好各种支持性政策，化压力为动力，化危机为转机，增强信心，抢抓机遇，扎实工作，努力推动经济社会又好又快发展。

　　综合考虑各种因素，今年我市经济社会发展的主要预期目标是：生产总值增长12%左右，人均生产总值增长11.8 %；单位生产总值能耗、主要污染物排放总量控制在省下达我市指标以内；工业总产值增长18%，农业总产值增长4%；社会消费品零售总额增长16%；全社会固定资产投资增长25%；实际吸收外商直接投资增长10%，外贸出口力争年内有所增长；地方财政一般预算收入增长16%；城镇登记失业率控制在3%以内；城镇居民人均可支配收入增长9%，农村居民人均纯收入增长8%；人口自然增长率控制在7‰左右。

　　为完成上述目标任务，要着力抓好以下八项工作：

　　一、 以扩大内需为重点，保持经济平稳较快增长

　　狠抓重大项目建设。集中力量抓好一批影响全局、支撑长远发展的重大项目。全力支持阳江核电站建设，确保广东华厦阳西电厂6月份正式投产，推动阳江抽水蓄能电站上马建设，着力打造广东重要电力能源基地。按期完成500千伏西蝶线等输电工程，进一步完善电网架构。推进云阳高速公路阳阳段、开阳高速公路阳江立交等一批重点项目建设。动工建设阳江港4个5万吨级码头泊位。抓好海陵大堤修复加固工程，确保4月中旬完成修复。积极配合做好广东西部沿海铁路项目建设前期工作，加大对年产量500万吨水泥生产基地等重大项目协调服务力度，力促尽快开工建设。借助国家加大投资的有利时机，积极争取一批重大项目列入国家和省重点项目范围，争取国家、省在重点项目的审批、用地、资金等方面给予更多的支持，推动全市经济加快发展。

　　积极扩大消费需求。千方百计增加居民收入，大力培育消费热点。加快发展连锁经营、物流配送等现代流通业，支持大型商业中心建设，优化消费环境。推进阳江港物流园区和五金产品物流中心、建材物流中心、卷烟配送中心、粮食物流中心、水产品物流中心、蔬菜水果物流中心“一园六中心”建设。落实“家电下乡”等消费政策，深入实施“万村千乡”市场工程，加强镇村农贸市场建设，支持大中型流通企业向小城镇延伸经营网络，扩大农村地区消费。

　　大力发展第三产业。坚持科学规划，有序开发，强化行业管理和宣传促销，着力打造国内外知名旅游休闲度假胜地。加强旅游基础设施建设，争取在观光旅游、度假休闲、特色旅游产品开发建设上取得新进展。着力推进阳东山水绿洲度假村、海陵岛海韵国际大酒店、阳春温泉度假村等十项旅游重点项目建设，培育特色旅游产品，提高旅游服务接待能力。以广东海上丝绸之路博物馆开馆为契机，发挥“南海Ⅰ号”效应，大力发展旅游文化产业集群。加强对房地产业的引导，适度开发大型楼盘，推进住宅产业化和集约化，促进房地产业健康有序发展。继续做大批发零售、餐饮服务等传统商贸业，培育发展商务会展、文化创意、电子商务、服务外包、现代中介、现代金融等新型城市经济业态，提升第三产业发展水平。

　　二、以壮大总量为重点，推动工业经济发展提速增效

　　提高自主创新能力。实施《阳江市推进自主创新行动纲要》，推进创新型阳江建设。用好用活中小企业发展专项基金，支持企业加强技术攻关和创新。进一步推进产学研合作，扶持有条件的企业组建省级工程技术研发中心和企业技术中心，提升企业技术创新能力。推动企业以五金刀剪省部产学研创新联盟为依托，积极组织五金刀剪产业共性关键技术的攻关，提高产业技术水平和市场竞争力。努力培育发展一批高新技术企业，力争年内有6家以上企业获得国家级高新技术企业认定。全面实施知识产权战略，推进省知识产权试点城市建设，争取年内培育省级知识产权优势企业2家。

　　加快发展临港工业。依托港口优势，大力发展以外向型、大物流为特征的临港工业。支持临港企业拓展市场，积极发展保税仓储业务，构筑现代港口物流体系，加快港口经济发展。着力推进嘉吉粮油二期工程等一批项目建设，加强一批在谈重化工业项目的跟踪落实，推动形成临港工业集群。

　　改造提升传统工业。以高新技术改造提升五金刀剪、纺织服装、食品加工、机电建材、家具制造等传统产业，推动传统产业创品牌、上规模、增效益。以国家刀剪及日用金属工具质量监督检验中心建设和省市共建五金刀剪先进制造业基地为契机，着力打造先进制造产业基地。

　　支持中小企业发展。落实和完善扶持中小企业发展的政策措施，加强中小企业服务平台建设，建立重点企业直通车服务制度，支持中小企业与大企业配套发展。加强对工业发展的协调服务，继续开展“工业企业集中服务月”活动，落实重点企业包扶责任制，及时为企业解决发展中的各种难题。全面清理涉企收费项目，扩充中小企业发展专项资金，加大财税政策扶持力度，帮扶企业渡过难关。财政参股组建中小企业担保机构，推进政企银企合作，加快中小企业融资服务体系建设，着力解决中小企业融资难问题，支持中小企业转型升级，促进中小企业加快发展。

　　努力扩大外贸出口。扶持优势区域特色产品和有自主知识产权、自主品牌、附加值高的产品出口，不断优化出口产品结构。积极利用国家相关出口优惠政策，多元化开拓出口市场。制定完善奖励措施，鼓励企业“走出去”，开展对外商贸洽谈，发展对外销售网络，扩大出口规模。扶助企业降低出口风险，鼓励使用出口信用保险工具，主动应对反倾销、反补贴等贸易壁垒。做好阳江港通用码头8号泊位对外开放验收工作，推进阳江港集装箱运输定期航班建设，尽快开通阳江港至深圳盐田港航线，降低出口企业的运输成本。加快口岸联检综合楼建设，改善通关环境。

　　三、以促进农民增收为重点，切实抓好“三农”工作

　　积极发展现代农业。稳定粮食生产，确保粮食供给安全。调整优化农业产业布局，大力发展南药、水果、蔬菜等特色经济作物，积极推动猪苗、蚕桑等基地建设。加快现代农业园区和农业标准化示范区建设，发展农产品加工业，培育壮大一批农业龙头企业，力争创建1家国家级、2家省级农业龙头企业。大力发展畜牧养殖业和现代林业，推进8万亩林业生态重点工程建设。充分发挥海洋资源优势，推进海洋综合开发，加快发展现代渔业。实施现代标准化鱼塘建设和健康养殖基地建设工程，努力完成1万亩标准化鱼塘建设。加快发展水产品加工流通业，打造广东省重要水产品加工出口基地。完善农业科技创新和服务体系建设。推动农业标准化生产，提高农产品质量安全水平。

　　完善农村基础设施。加大对农村基础设施建设和社会事业发展的投入，加快推进公共资源向农村倾斜、公共设施向农村延伸、公共服务向农村覆盖。抓好400公里农村公路建设，不断完善农村路网。高标准抓好海堤达标建设，全市万亩以上海堤按五十年一遇标准年内全部开工。完成3座中型水库和24座小型水库的除险加固任务；积极推进漠阳江两岸和中小流域整治。加快中低产田改造，重点抓好市级1万亩、县级7000亩两个标准农田示范区建设。加快东平国家中心渔港建设，完成沙扒国家一级渔港建设任务，推动溪头、对岸渔港创建国家级渔港。加强农村饮水安全工程和农村防灾减灾工程建设，扎实开展“宜居村庄”创建活动，推进社会主义新农村建设。

　　努力促进农民增收。全面落实各项强农惠农措施，完善财政支农资金稳定增长机制，保障农民收入稳定增长。稳定和完善农村基本经营制度，加快推进农村土地承包经营权流转，鼓励农民流转土地承包经营权，增加农民的土地收益。引导开展多种形式的生态、休闲、观光农业经营活动，加快发展农民专业合作社，扶持农业产业化经营，鼓励发展农产品加工，挖掘农业内部增收潜力。拓展农村非农就业空间，鼓励农民就近就地就业，努力增加农民的工资性收入。落实农民工返乡创业扶持政策，在贷款发放、税费减免、工商登记、信息咨询等方面提供支持，拓宽农民增收渠道。稳步推进城乡经济社会发展一体化。

　　四、以加快省示范性产业转移园建设为重点，强力推进“双转移”工作

　　加快融入珠三角。深入学习贯彻《珠江三角洲地区改革发展规划纲要》，主动融入珠三角，依托珠三角，服务珠三角，积极接受珠三角的辐射和带动，承接珠三角的产业转移。加强与珠三角在产业体系和交通网络等方面的对接，加快融入以珠三角为中心的资源互补、产业关联、梯度发展的多层次产业圈，与珠三角形成基础设施对接共享、产业发展合作共赢的新格局，不断提高与珠三角的合作水平。

　　着力加快产业转移园建设。以广州(阳江)产业转移工业园开发建设作为经济发展的重要载体，高起点做好园区的发展规划，合理设置园区功能分区，引导企业向园区集聚。集中政府可调控的生产要素，向工业园区倾斜，加快完善园区各项基础设施。用好用活省竞争性扶持资金，拓宽园区建设融资渠道，提升其对项目、资金、人才、技术等要素的聚集能力。制订完善优惠的政策措施，创造园区工业用地、用水、用电价格优势，推动一批工业项目上马建设。统筹发展中山火炬(阳西)等产业转移工业园的建设，全面提升我市承接产业转移的竞争力，把我市打造成承接产业转移的示范地。

　　加大招商引资力度。继续开展驻点招商活动，主动承接产业转移，努力引进世界500强及国内大型知名企业到我市投资，提高招商引资的规模和质量。结合我市资源、产业优势，着力引导外资投向临港工业、先进制造业、文化旅游业、农海产品深加工业、现代物流业和服务业，优化利用外资结构。积极鼓励现有大型外资企业增资扩产，吸引业务相关或上下游配套企业。加强投资服务管理，为客商提供贴身贴心服务，做到“将方便让给客商，将麻烦留给自己”，努力降低营商成本，增强投资吸引力。强化签约项目的跟踪服务，提高签约项目的合同履约率、资金到位率、项目开工率。

　　推进农村劳动力转移就业。认真做好农村劳动力转移就业总体规划，落实促进劳动力培训转移就业相关政策。大力开展农村劳动力培训，重点在建立县镇农村劳动力培训基地和完善劳务输出服务网络上下功夫，加快推进农村富余劳动力转移就业。全年计划培训劳动力4.5万人次，新增农村劳动力转移就业6.8万人。

　　五、以建设宜居城乡为重点，全面提升城乡规划建设管理水平

　　强化城乡规划。按照建设宜居创业滨海新城的目标要求，加强规划的编制、管理和实施，优化城镇体系规划和城乡功能布局。组织编制好城市分区规划，重点抓好产业转移工业园、三江岛等的规划编制工作，推进“一河两岸”、“城南新区”和共青湖核电生活后勤科研基地等重点项目规划的实施。贯彻实施《城乡规划法》，继续抓好城镇总体规划的修编和控制性详细规划的编制工作，全面完成全市12个中心镇的总体规划修编。

　　统筹城乡建设。加快阳阳铁路延伸线、S369线春湾至罗阳段改建工程和市区二环南路、东门南路、高凉路、开阳高速公路阳江立交联络线等一批公路、市政道路的建设，确保年内竣工通车。推进沿海高速公路联络线白沙至程村段、阳西县织至丰头港公路等一批公路的开工建设。尽快开工建设新江东路、马曹路西段等一批市政道路，进一步拉开城市建设框架。结合城市防洪工程建设，加快推进“一河两岸”改造工程。

　　完善城市管理。树立经营城市理念，引导社会资金参与城镇基础设施建设，逐步引入竞争机制，强化市政管理。进一步完善市区建设管理体制，加大城市管理和执法力度，大力整治“六乱”和占道经营等行为，规范和美化户外广告，美化城市环境，改善环卫设施，提高城市管理水平。进一步建立健全土地储备制度，全面严格清理闲置土地，盘活土地资源，推进节约集约用地，增强政府对城市土地开发和利用的调控能力。

　　加强生态环境保护。认真落实节能减排工作责任制，严格执行建设项目环境准入管理和“三同时”制度，鼓励发展低能耗、高附加值的产业，从严限制高消耗、高污染项目。严格实施环境保护规划，加大环保执法力度。推进城市污水收费改革，加快闸坡生活污水处理厂建设步伐，年内上马建成城南新区、高新区、银岭工业园等污水处理厂。继续推进重点污染源在线监控设施建设，稳妥推进岗列那格五金电镀城搬迁，规划建设工业固体废物处理中心。扎实开展创建绿色城镇、绿色学校、绿色企业、绿色社区和生态示范区等活动，在加快发展的同时保护阳江的绿水青山。

　　六、以创新体制机制为重点，继续深化各项改革

　　积极推进行政体制改革。按照省的部署和大部门体制的要求，坚持精简、统一、效能的原则，稳妥推进政府机构改革，合理配置部门职能，理顺部门职责关系。加强行政执法部门建设，完善行政执法机制。开展第四轮行政审批制度改革。稳步推进事业单位分类改革。

　　加快国有企业改革。建立健全国有资产的监督、审核、处置机制，确保国有资产保值增值。有序推进市属国有企业改革，加快劣势企业退出市场。从今年起政府每年安排3000万元作为国企改革专项资金，盘活存量资产，处理好国企改革的遗留问题，力争用2-3年时间全面完成国企改革工作。支持国有企业引入战略投资者，加快产权多元化进程，优化经营机制。

　　深化投融资、财税和金融体制改革。创新投融资体制，重点推行BOT、BT、项目代建制、收费质押等新型项目融资和组织方式，推进政府公共资源特许经营改革，试行城市公共资源开发、使用、经营公开招标，增加公共资源收益。完善财税管理机制，贯彻落实国家和省的各项税收政策，依法征税。建立健全金融服务机构，加强金融工作的协调服务。设立新型金融机构，搭建融资平台。深化农信社改革，发挥其在新农村建设中的信贷主渠道作用。稳步推进价格和收费等各项改革。

　　完善现代市场体系。进一步健全资本、产权、土地、人力资源等要素市场。规范发展行业协会和市场中介组织。创新市场监督模式，严厉打击制假售假、走私贩私、商业欺诈、传销等违法行为。加快社会信用体系建设，健全信用监督和失信惩戒制度。

　　七、以改善民生为重点，着力构建和谐阳江

　　优先发展教育。多渠道筹措资金，加大教育投入。巩固提高义务教育水平，重点抓好100所义务教育阶段学校规范化建设，年底前解决全市517名农村中小学代课教师问题。加快普及高中阶段教育，扩建10所高中阶段学校，新增学位近万个，积极筹备迁建市第一中学，阳西县第二中学今年9月要建成招生，确保全市高中阶段教育毛入学率达到68%。大力发展职业技术教育，建设一批重点职业学校、重点专业和实训中心，加快市技工学校、市第一职业技术学校扩建扩招工作。优化高等教育，确保阳江职业技术学院通过教育部人才培养工作水平评估。规范发展民办教育，逐步解决外来务工人员子女教育问题。重视学前教育，关心特殊教育。加强教师职业道德教育，提高教师队伍整体素质。加强教学管理，强化素质教育，提高教育质量，办好人民满意教育。

　　不断提高就业和社会保障水平。健全就业工作目标责任制，实施更加积极的就业政策，积极促进城镇新增劳动力、高校毕业生、就业困难群体和下岗失业人员等各类劳动者实现就业。城镇新增就业人员4万人。抓好地税全责征收社保费，妥善解决行政事业单位社保遗留问题，推进社会保险扩面征缴。加快养老保险市级统筹。扎实推进城镇职工、城镇居民基本医疗保险和新型农村合作医疗工作，加快实现全民医保。实施被征地农民养老保险试行办法，全面铺开被征地农民养老保险制度。抓好低保应保尽保工作，落实五保供养资金，确保集中供养标准达200元/人·月、分散供养标准达150元/人·月。加强市红十字会和慈善会建设，筹建省红十字会粤西片备灾救灾中心，建立健全社会救助体系，做好因病致贫、因贫失学、因残失业、因灾受困家庭和人员的救助援助工作。加快廉租住房建设，逐步解决困难群众“住房难”问题。

　　加快医疗卫生事业发展。推进医疗卫生体制改革试点，促进基本公共卫生服务均等化。整合各类精神卫生资源，建立市精神卫生防治机构。加强社区卫生服务机构建设，改造建设10间社区卫生服务中心。加快镇、村卫生院（站）综合改革，促进农村卫生事业健康发展。推进中医药振兴工作，新建市中医医院。加强医疗机构能力建设，提高医疗服务水平和救治能力。继续加强无偿献血和传染病防治等工作。

　　大力发展文化事业。加快广东海上丝绸之路博物馆工程建设步伐，力争年中建成开馆。继续推进文化名城“五个十”工程建设。加快推进市粤剧团、歌舞团的改革。加强文物、非物质文化遗产保护和文化市场管理。完成第三次全国文物普查工作任务。深入挖掘开发“海上丝绸之路”、冼冯遗迹等历史文化资源，创作和生产具有阳江特色的文化产品和艺术精品。大力发展广播、影视、出版、创意、娱乐等新兴文化产业，培育文化龙头企业。

　　维护社会和谐稳定。重视解决群众关注的热点难点问题。强化市场监管，保障粮油肉等基本生活必需品和其他紧缺商品的有效供给，努力保持重要消费品和服务价格基本稳定。加强食品药品监管，保障群众的健康安全。完善安全生产责任制，加大消防设施的投入，加强安全生产监管和执法工作，严肃事故查处和责任追究，保持安全生产形势持续稳定。建立健全各种预警和应急机制，强化动物防疫体系建设，加强对自然灾害和气候变化的监测、预报、预警和防控，及时应对和处置突发公共事件。建立健全信访工作长效机制，深入推进“县委书记大接访”、“行风热线”等活动，积极引导群众合理合法反映诉求。加大人民调解和社区矫正力度，做好社会矛盾纠纷排查调处化解工作。完善社会治安防控体系，完成视频监控系统二期工程建设。依法严厉打击各类违法犯罪活动，加大破案力度，大力整治社会治安突出问题，进一步压减治安和刑事案件发生。加大法制宣传教育力度，推进和谐社区创建活动。切实维护弱势群体的合法权益，努力维护社会稳定。

　　加快发展其他各项社会事业。做好人才工作，完善人才激励机制，大力引进各类人才。落实计生工作责任制，综合治理出生人口性别比偏高问题，稳定低生育水平，提高出生人口素质。加快发展体育事业，推进全民健身运动，加快市体育运动学校、市水上活动中心建设。重视科普工作，加快市科技馆内部装修布展和开馆。积极推进广播电视事业发展。加大档案馆舍建设和档案资源抢救、保护、开发力度。加强国防动员、民兵预备役、拥军优属工作。全面完成全国第二次经济普查工作任务。加快气象公园和海洋气象监测网建设，完善气象防灾减灾体系。继续做好妇女儿童、外事侨务、民族宗教、防震减灾、人防、打私、市志、残疾人、关心下一代等工作，推动社会全面进步。

　　八、以提高行政效能为重点，加强服务型政府建设

　　新的形势和任务对政府工作提出了更高的要求。我们要以建设服务型政府为目标，着力加强自身建设，努力使政府职能转变有新突破，科学民主决策和依法行政有新进展，服务能力和行政效率有新提高。

　　提高科学决策能力。牢固确立科学决策、民主决策的理念，坚持公众参与、专家论证和政府决策相结合的决策机制，提高决策的科学化、民主化水平；坚持问政于民、问需于民、问计于民，深入实际调查研究，做到重大决策依民而定，工作措施应民而出，安危冷暖唯民而系，使提出的发展思路、工作部署、政策措施更加符合发展规律，更加符合阳江实际，更加富有成效。

　　提高开拓创新能力。围绕建设学习型政府，切实加强政策理论和社会管理知识的学习，不断提高攻坚克难、改革创新的能力。围绕推进科学发展，勇于走出传统的思维模式，积极改革不符合科学发展要求的体制机制，创造性开展工作，始终以创新之举破解发展难题。围绕增强实践能力，善于从国内外发展大势中捕捉和把握机遇，善于在逆境中发现和培育有利因素，抢占发展先机，实现加快发展。

　　提高服务群众能力。把服务作为政府履行职责和行使职能的基本要求，不断增强服务能力，改进服务方式，提高服务水平。加强公务员队伍建设，提高为基层和群众办实事、办好事的本领。积极推进政务服务方式创新，拓展网上受理、并联审批和一站式服务，提高服务质量与效率，更好地为企业和群众服务。加强电子政务建设，充分发挥网络服务功能，提高政府工作效率。加大政务环境整治力度，坚决从严查处影响投资环境的违法违纪行为，切实维护企业和群众的合法权益。

　　提高真抓实干能力。坚持把抓落实摆在政府工作首位，建立岗位责任制、重点工作责任制和督查制度，全面推行行政问责制，保证政府对人民群众的每一项承诺都落到实处。进一步规范面向镇（街道）的检查考核活动，精简会议，压减文件，把更多的时间和精力放在调查研究和抓督促落实上。积极争取上级支持，主动谋划推进工作。健全完善各种行政管理制度，实施精细化管理，优化工作流程，努力以更高的标准、更好的服务、更快的效率抓好工作的落实，使政府施政行为更有序、高效。

　　提高依法行政能力。坚持依法行政、从严治政。严格执行人大及其常委会的决议、决定，自觉接受人大及其常委会的法律监督和工作监督。加强与人民政协的协商，自觉接受人民政协的民主监督。认真听取各民主党派、工商联和无党派人士的意见和建议。广泛征集群众建议，接受社会公众和新闻舆论监督。加大办理人大代表议案、建议和政协提案的力度，提高办理质量。严格按照法定权限和程序行使权力、履行职责，规范行政行为，不断提高依法行政水平。加强政府廉政勤政建设，继续开展机关作风评议和行风评议活动，坚决纠正侵害群众利益的不正之风，树立政府良好形象。

　　各位代表，新的一年，充满新的机遇，孕育新的希望。让我们在省委、省政府和市委的领导下，高举中国特色社会主义伟大旗帜，深入贯彻落实科学发展观，解放思想、振奋精神、团结一致、创新实干，为把阳江建设成为三次产业协调发展的新型工业化城市而努力奋斗！
